# Hippocampal developmental vulnerability to methylmercury extends into prepubescence

**DOI:** 10.3389/fnins.2015.00150

**Published:** 2015-05-12

**Authors:** Maryann Obiorah, Elizabeth McCandlish, Brian Buckley, Emanuel DiCicco-Bloom

**Affiliations:** ^1^Department of Neuroscience and Cell Biology, Rutgers Robert Wood Johnson Medical School, Rutgers The State University of New JerseyPiscataway, NJ, USA; ^2^Environmental and Occupational Health Sciences Institute, Rutgers The State University of New JerseyPiscataway, NJ, USA; ^3^Department of Pediatrics, Rutgers Robert Wood Johnson Medical School, Rutgers The State University of New JerseyNew Brunswick, NJ, USA

**Keywords:** neurogenesis, methylmercury, hippocampus, neural stem cell, development

## Abstract

The developing brain is sensitive to environmental toxicants such as methylmercury (MeHg), to which humans are exposed via contaminated seafood. Prenatal exposure in children is associated with learning, memory and IQ deficits, which can result from hippocampal dysfunction. To explore underlying mechanisms, we have used the postnatal day (P7) rat to model the third trimester of human gestation. We previously showed that a single low exposure (0.6 μg/gbw) that approaches human exposure reduced hippocampal neurogenesis in the dentate gyrus (DG) 24 h later, producing later proliferation and memory deficits in adolescence. Yet, the vulnerable stem cell population and period of developmental vulnerability remain undefined. In this study, we find that P7 exposure of stem cells has long-term consequences for adolescent neurogenesis. It reduced the number of mitotic S-phase cells (BrdU), especially those in the highly proliferative Tbr2+ population, and immature neurons (Doublecortin) in adolescence, suggesting partial depletion of the later stem cell pool. To define developmental vulnerability to MeHg in prepubescent (P14) and adolescent (P21) rats, we examined acute 24 h effects of MeHg exposure on mitosis and apoptosis. We found that low exposure did not adversely impact neurogenesis at either age, but that a higher exposure (5 μg/gbw) at P14 reduced the total number of neural stem cells (Sox2+) by 23% and BrdU+ cells by 26% in the DG hilus, suggesting that vulnerability diminishes with age. To determine whether these effects reflect changes in MeHg transfer across the blood brain barrier (BBB), we assessed Hg content in the hippocampus after peripheral injection and found that similar levels (~800 ng/gm) were obtained at 24 h at both P14 and P21, declining in parallel, suggesting that changes in vulnerability depend more on local tissue and cellular mechanisms. Together, we show that MeHg vulnerability declines with age, and that early exposure impairs later neurogenesis in older juveniles.

## Introduction

Methylmercury (MeHg), an organic form of mercury (Hg), is a common neurotoxicant found in contaminated fish and shellfish. Consumption of these foods is the main route of human exposure (NAS, 2000). In humans, MeHg acts as a teratogen and can cross the placenta and the blood brain barrier (BBB), causing damage to brain regions such as cerebral cortex, cerebellum, and hippocampus. For decades, MeHg was documented to cause neurological abnormalities in adults such as paresthesia, ataxia, and dysarthria. However, not until the late 20th century did scientists observe MeHg's effects on the extremely vulnerable developing brain during two major poisoning events in Japan and Iraq. In both cases, adults presented neurological symptoms only following a latency period. Surprisingly, however, children born to women with minimal or no poisoning symptoms also displayed severe neurological defects such as mental retardation, seizures, and blindness, suggesting the developing brain had greater sensitivity to MeHg that had crossed the placenta and fetal BBB (Matsumoto et al., 1965; Amin-Zaki et al., 1976; Choi et al., 1978; Harada et al., 1999; Philbert et al., 2000; Weiss et al., 2002). In addition to these high level-poisoning events, other population studies suggested that the developing brain was also vulnerable to even more common exposures attained through the maternal diet. Long-term epidemiological studies on children from frequent fish-eating populations found variable effects of prenatal low-level MeHg exposure (Myers et al., 1995b; Grandjean et al., 1997; Debes et al., 2006; Davidson et al., 2011). For example, New Zealander and Faroese children born to mothers who regularly consumed fish during pregnancy had deficits in IQ, motor skills, cognitive abilities, and language development (Kjellstrom et al., 1986, 1989; Grandjean et al., 1997; Crump et al., 1998; Debes et al., 2006). Yet, in Seychellois children, maternal MeHg exposure did not appear to be associated with neurological deficits (Myers et al., 1995a,b,c; Davidson et al., 2011). These variable outcomes suggest that the extent of perceived MeHg insult during development depends on the frequency (seasonal or year-round) of fish consumption, level of contamination, presence of other pollutants, methods for inferring fetal exposure (MeHg in mother's hair or umbilical cord blood), sensitivity of neurological tests and overall diet of the populations (Clarkson and Magos, 2006). The manifestation of cognitive deficits (especially learning and memory) in childhood after prenatal MeHg exposure suggests that early insults cause cellular alterations that might result in later hippocampal dysfunction. Evidence from these studies highlights a period of developmental vulnerability in the hippocampus that begins prenatally, but when (or if) it ends is not known.

Given the effects of MeHg exposure on learning and memory, it is reasonable to focus on neurogenesis that occurs in the dentate gyrus (DG) of the hippocampus throughout development into adulthood (Deng et al., 2010). In the subgranular zone (SGZ) of the DG, neural stem cells (NSCs) have a radial glia-like morphology and express astrocytic marker glial fibrillary acidic protein (GFAP), intermediate filament nestin, and transcription factor Sox2. They undergo intermittently asymmetric cell division. Their progeny, termed intermediate progenitor cells, cease GFAP expression and undergo frequent symmetric divisions that comprise most of the DG dividing cell population. These cells also express the transcription factor Tbr2 (T-box brain gene 2). As they begin to commit to the neuronal lineage, they lose Tbr2 and begin to express doublecortin (Dcx), a neuron-specific microtubule-associated protein. Dcx expression continues into the neuroblast stage, during which cells exit the cell cycle, migrate into the granule cell layer (GCL) of the DG and differentiate into immature neurons that express Prox1. After a couple of weeks, NeuN- and calbindin-expressing mature granule neurons are formed and integrate into the existing neuronal network that modulates learning and memory (Kempermann et al., 2004; Encinas et al., 2006; Hodge et al., 2008). The hippocampus is unique in that a major portion of its neuronal development in rodents occurs postnatally in the DG. Postnatal neurogenesis, occurring from birth until just before puberty, takes place in the hilus while adult neurogenesis occurs only in the two/three-cell layered SGZ of the DG (Altman and Bayer, 1990; Eisch et al., 2008).

In rats, the peak of postnatal neurogenesis (postnatal day 7–P7) corresponds to human hippocampal development occurring during the third trimester of gestation. In our previous studies using P7 rats, we used a single MeHg exposure model coupled with cell-specific immunohistochemical analysis in the DG to clarify the temporal schedule of cellular events in the hippocampus (Burke et al., 2006; Falluel-Morel et al., 2007; Sokolowski et al., 2011, 2013). We used two exposures in our paradigm: 5 μg/g, a classical exposure chosen because of acute effects on the mitotic spindle without signs of overt poisoning (Rodier et al., 1984) and 0.6 μg/g (which approximates human exposures) (Myers et al., 1995b; Falluel-Morel et al., 2012; Sokolowski et al., 2013). Twenty-four hours after exposure, MeHg induced cell cycle arrest during the G1/S phase transition, due to cyclin E degradation, and subsequent cell death (Burke et al., 2006; Falluel-Morel et al., 2007). NSCs were considered to be the vulnerable population as they underwent apoptosis upon acute exposure (Sokolowski et al., 2013). This acute NSC loss was associated with a later reduction of DG granule neurons (~27%) by early adolescence (P21), whose deficiency might have contributed to the deficits in spatial learning and memory (Morris Water Maze task) at P35 (Falluel-Morel et al., 2007; Sokolowski et al., 2013). Alternatively, cognitive functions might have been affected by changes in the process of neurogenesis itself, because we found that mitotic S-phase cells were reduced by 24% at P21 (Sokolowski et al., 2013). We do not know whether this decrease in proliferation was due to a sustained loss of NSCs that followed the acute injury, or a change in their later proliferative activity, an issue we now address. Most immature neurons born through neurogenesis die within the first week of their birth, and it is possible that MeHg can impact them during this critical period of survival (Sierra et al., 2010) since MeHg can still be detected in the hippocampus at P21 (after a 5 μg/g P7 exposure) (Sokolowski et al., 2013). In this study we will extend the analysis of this paradigm to identify the affected cell compartments in the adolescent neurogenic cascade.

Deficits in hippocampal-dependent behavioral tasks might be a sign of impaired neurogenesis (Deng et al., 2010). Since prenatal MeHg exposure is implicated in cognitive deficits in school-age children (Kjellstrom et al., 1986, 1989; Grandjean et al., 1997; Crump et al., 1998; Debes et al., 2006), it is possible that hippocampal neurogenesis is negatively impacted only during periods of developmental vulnerability. However, the length of this period in the developing rat DG is not known. Thus, we now use our single-exposure paradigm in rats to answer two questions: First, what is the effect of an early exposure (P7) on adolescent neurogenesis (P21), and is there a specific cell compartment affected? Next, when does the period of developmental vulnerability to acute MeHg toxicity end? Since the rate of postnatal neurogenesis in the rat is highest during the first 3 weeks of life (Kempermann, 2011), we have studied the temporal extent of this vulnerability in prepubescent (P14) and adolescent (P21) rats. Knowing more about the effects of perinatal MeHg exposure may help us identify critical pathways that contribute to neurological deficits and may be useful to inform dietary guidelines for women during pregnancy.

## Materials and methods

### Ethics statement

All animal procedures and experimental protocols were approved by the Robert Wood Johnson Medical School Institutional Animal Care and Utilization Committee (IACUC) and conformed to NIH Guidelines for animal use. Experiments were designed so as to use a minimum number of animals and minimize discomfort.

### Experimental animals

Male Sprague-Dawley rat pups were used for all experiments in this study. Rat litters with mothers were purchased from Hilltop Lab Animals (Philadelphia, PA). Hilltop provides a litter of 12 cross-fostered male pups with a nursing mother. Upon arrival in the temperature- and light-controlled animal care facility, animals were allowed to acclimatize (receiving food and water *ad libitum*) for at least 3 days before undergoing experimental procedures. For both acute (P14, P21) and long-term (P7) exposure experiments, littermates were subcutaneously (sc) injected with 100 μL volume of vehicle (phosphate-buffered saline—PBS) or MeHg (0.6 or 5 μg/gbw), forming a within-litter dosing design (Sokolowski et al., 2013). Sample size per experimental group was based on the total number of pups in a litter divided by three, (i.e., 4 pups per group in a 12-pup litter). One litter comprised one experiment, and each experiment was performed at least three times. Pups remained with their cross fostering mothers throughout the experiment. Although maternal care is known to affect developmental neurogenesis, forming all experimental groups within each litter reduces the likelihood that maternal care contributes to group bias. This design restricts variability within each pup to being caused by individual biological response and injection efficacy, and not by specific litter. Therefore, sample size (*n*) was based on the number of pups. Instead of administering MeHg via gavage, sc injection was used to ensure equal exposures, as we have done for previous quantitative kinetic studies (Burke et al., 2006; Sokolowski et al., 2013). Furthermore, gastrointestinal MeHg absorption may vary depending on gut enzymes and other contents (which may differ in developing animals).

### Methylmercury

Methylmercury chloride (CH3HgCl) was purchased from Sigma-Aldrich (St Louis, MO). A 1.5-mg/mL stock solution in 0.1 M PBS was prepared by agitation in sealed glass bottles for 2 h immediately before use.

### BrdU and EdU administration and detection

To measure proliferating cells during S-phase, P15, P22, and P23 pups received a single intraperitoneal (ip) injection of 50 mg/kg Bromodeoxyuridine (BrdU) or 5-ethynyl-2′-deoxyuridine (EdU) 2 h before sacrifice, after which brains were processed for immunohistochemistry. Our earlier studies were conducted using BrdU. However, to reduce the potential for confounding variables caused by classical immunohistochemical BrdU detection protocols such as antigen retrieval and HCl denaturation, we used EdU and the Click-iT® EdU Imaging Assay Kit (Life Technologies, NY, USA) for detection in subsequent studies. To ensure that EdU is comparable with BrdU in labeling S-phase cells in the rat hilus, we injected P7 rats with both (BrdU and EdU) and used respective immunohistochemical detection techniques to visualize labeled cells. We found that 98% of BrdU+ cells were immunopositive for EdU.

### Tissue collection and preparation

Pups were gently restrained while receiving ip injection of anesthetic (Ketamine/Xylazine cocktail—75 mg/kg and 10 mg/kg, respectively). Animals were perfused with 15–25 ml of 0.9% NaCl followed by 15–25 ml of 4% paraformaldehyde (PFA) in 0.1 mol/L PBS. Brains were dissected and postfixed in 4% PFA for 16 h at 4°C, cryoprotected in 30% sucrose-PBS for at least 3 days, and quickly frozen in OCT medium (Tissue-Tek, Sakura, Tokyo, Japan). Tissue blocks were stored at −80°C until sectioning. Coronal frozen sections (12–20 μm thickness, depending on the age of the animal) were made using a cryostat (Leica, Heidelberg, Germany) in a 1:10 series and stored at −20°C until immunostaining.

### Immunohistochemistry

Sections were washed 3 times in PBS before proceeding with immunostaining. BrdU detection required antigen retrieval (steaming at 95°C in 0.01 mol/L citrate buffer for 10 min) and incubation in 2N HCl (30 min) before antibody incubation. For all other stains (including double staining), sections underwent antigen retrieval, then incubation in 33% normal serum in PBS (1 h) and primary antibody (overnight) at room temperature in 0.3% Triton X-100 and 1% normal serum in PBS. The primary antibodies used were: mouse monoclonal anti-BrdU (1:100; Becton-Dickinson, San Jose, CA), rabbit monoclonal anti-cleaved Caspase 3 (1:300; Cell Signaling, Beverly, MA), rabbit polyclonal anti-Ki67 (1:500; Abcam, ab15580, Cambridge, England), rabbit polyclonal anti-Sox2 (1:1000; Abcam, ab97959), rabbit polyclonal anti-Tbr2 (1:300, Abcam, ab115986) and guinea pig polyclonal anti-Doublecortin (1:1000; Millipore, ab5910, Temecula, CA). For fluorescent staining, the secondary antibodies used were: Alexa Goat anti-Mouse 488, Alexa Goat anti-Rabbit 594, and Alexa Goat anti-Guinea pig 594 (Molecular Probes, Eugene, OR).

### Image analysis

Six to twelve animals/group, obtained from three separate experiments, were analyzed. Sections were visualized on an Axio Imager.M2 microscope (Zeiss, Thornwood, NY). Positive cells were counted on unilateral hippocampi from 4 or 5 sections/animal. These sections were derived from every 10th section of the middle third of the hippocampus (between Bregma −1.34 and −2.54 mm, a region that is most proliferative at this age, Wagner et al., 1999; Cheng et al., 2002). The first cell layer of the SGZ was included in the GCL, while the other SGZ cells were counted as part of the hilus. All positive cells present in the selected sections and regions were counted. Cells were counted blind to experimental group at high magnification (at least 20× objective) using Image J software (Rasband, 1997–2012).

#### Doublecortin analysis

Due to the large number of Doublecortin (Dcx)-positive cells, the numbers of cells/section were counted via unbiased stereology using the Optical Fractionator of MicroBrightField (MBF) Stereo Investigator system (Williston, VT, USA). Outlines of the DG were drawn at low magnification, and somas were counted under 40× objective (400× magnification). Every 10th section through the middle third of the hippocampus was analyzed, and data were obtained from 5 sections (12 μm each) per animal. Quantification of cells observed on unilateral hippocampi was performed for 6–12 animals per group, obtained from three independent experiments.

### Inductively coupled plasma mass spectrometry

P14 and P21 rat pups received a single sc injection of vehicle (PBS) or MeHg (0.6 or 5 μg/g body weight). At their respective time points, animals (*n* = 3 per group and time point) were perfused with 0.9% NaCl to remove blood from tissues. Whole hippocampi were collected for analysis at 2, 24, 48 h (P21 pups only) 2 and 4 weeks post-exposure. These time points were chosen for their relevance to our prior mechanistic and cell characterization studies (2–24 h), stereological measures (2 weeks), and behavioral analysis (4 weeks) (Sokolowski et al., 2011). In older rats, expression of neutral amino acid transporters (which are known conduits of MeHg) in the BBB is reduced (Cornford et al., 1982; Simmons-Willis et al., 2002; Liddelow et al., 2012). Therefore, we included an additional time point (48 h) in P21-exposed rats to see if MeHg entry into the hippocampus is delayed. Three animals per MeHg group per time point were used, and the hippocampi of control animals from each time point were pooled into one sample. Hippocampal tissues (<150 mg wet weight) were placed into a conical tube. To each tube, 0.25 mL of concentrated nitric acid (EMD Omni-Trace Ultra High Purity, VWR Scientific) was added and the samples were allowed to react during active sonication. They were subsequently digested using a MARSX microwave sample digester (CEM Corp., Mathews NC). Final concentrations were diluted to 5% acid using DI water (MilliQ Ultrapure De- ionized, Millipore Corp., Billerica, MA) for a total volume of 7 mL. Internal standards and controls included: acid blank, acid spike, matrix blank and matrix spike. The acid blank had no tissue and no Hg added, while the acid spike had a known amount of Hg added. The matrix spike was an untreated piece of tissue with a known amount of Hg added. Samples were analyzed for Hg using a 53 (Thermo Electron, MA) inductively coupled plasma mass spectrometer (ICPMS). The m/z of 202 was used for quantitation, while m/z of 199, 200, and 201 were also observed as a QC measure.

### Statistical evaluation

Unpaired Student's *t*-tests were used to analyze all experiments. Since the goal of these experiments was to determine whether MeHg has negative effects at ages older than P7, we compared exposures to PBS vehicle alone. We were not asking whether one exposure was different than another, which analysis would necessarily require ANOVA for multiple group comparisons. All analyses were performed using GraphPad Prism 6.0 software. Probabilities of less than 0.05 were regarded as statistically significant. Data were expressed as arithmetic means ± SEM for all experimental measurements.

## Results

The following experiments extend in two directions previous results obtained using acute MeHg exposure in P7 rats. First, while previous study of P7 exposure found acute NSC cell cycle arrest and cell death as well as later adolescent deficits in proliferation, the cellular mechanisms remain undefined. Since the acute effects of MeHg on P7 NSC mitosis and apoptosis are extensively reported (Burke et al., 2006; Falluel-Morel et al., 2007, 2012; Sokolowski et al., 2011, 2013), we now only explore later consequences of early exposure on adolescent (P21) stem cell pools, in the following section. Second, we then determine the period of vulnerability of NSCs by looking at later ages, P14 and P21, using the same acute exposure paradigms as previously reported for P7. We also relate developmental changes in vulnerability to the efficacy of mercury transfer across the blood-brain-barrier (BBB) with advancing age.

### Perinatal MeHg exposure leads to reduced neurogenesis at adolescence

**Figure 1:**
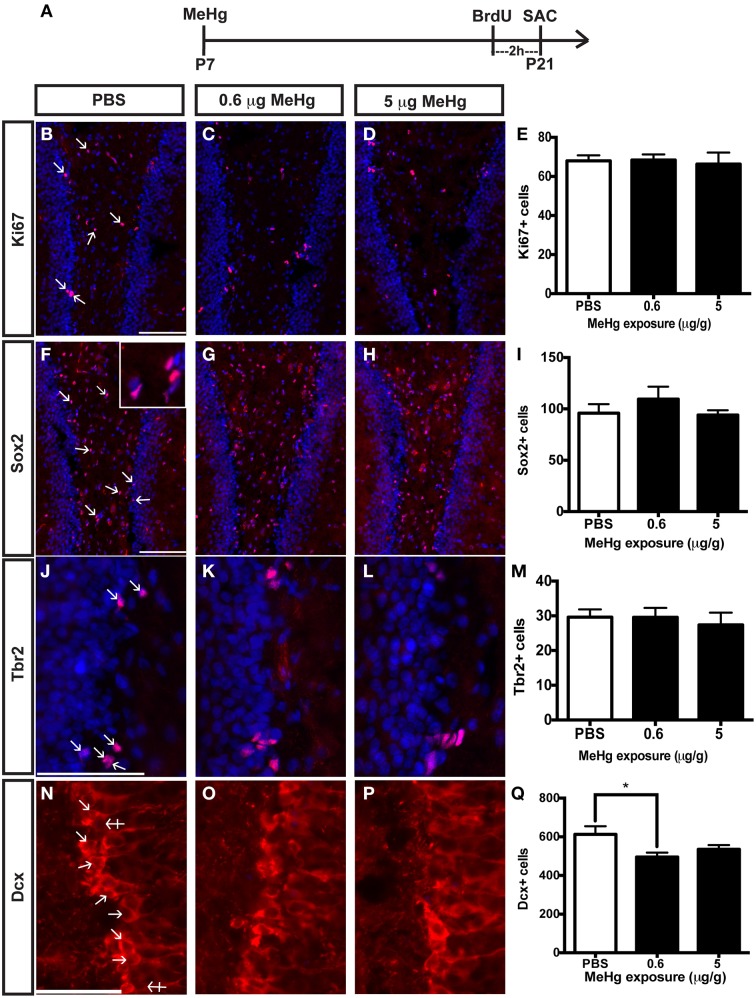
**Early (P7) MeHg exposure leads to an immature neuron deficit in the adolescent (P21) hippocampus. (A)** Experimental timeline. Five coronal sections of the hippocampus per brain were counted. **(B–D)** Ki67 (red) staining in the hilus of the dentate gyrus (DG). Arrows indicate positive cells. Nuclei are labeled with DAPI (blue). **(E)** Quantification of the average number of Ki67+ cells per section per animal in the hilus. Animals: *n* = 6–7 per group. Each bar represents mean ± SEM. Scale bars: 100 μm. **(F–H)** Sox2 (red) staining in the hilus of the DG. **(I)** Quantification of the average number of Sox2+ cells per section per animal in the hilus. Animals: *n* = 6–7 per group. Scale bars: 100 μm. (**J–L)** Tbr2 (red) staining in the hilus of the DG. **(M)** Quantification of the average number of Tbr2+ cells per section per animal in the DG. Animals: *n* = 5–7 per group. Scale bars: 100 μm. **(N–P)** Dcx staining in the GCL of the DG. Crossed arrows indicate negative cells. **(Q)** Quantification of the average number of Dcx+ cells per section per animal in the DG. Animals: *n* = 9–10 per group. Scale bars: 50 μm. ^*^p < 0.05.

The current experiments focus only on P21 rats that have been exposed to MeHg at P7 so we can determine the cellular mechanisms that contribute to adolescent deficits. We previously defined the molecular and cellular events that occurred upon acute MeHg exposure in P7 rats, and found that within 24 h there was a >30% reduction in mitotic S phase cells (BrdU+) in the DG hilus and >50% reduction in the size of the Sox2+ NSC population, suggesting stem cells were particularly vulnerable (Sokolowski et al., 2013). MeHg exposure led to reductions in cell cycle regulator cyclin E, as well as induction of mitochondrial-dependent apoptosis (Burke et al., 2006; Sokolowski et al., 2011). Indeed, MeHg exposure elicited a 3–5-fold increase in caspase-3+ cells in the hilus, with >75% labeling with stem cell marker, nestin, and 32% with Sox2, which led to later cell deficits in the hilus and GCL by adolescence (Falluel-Morel et al., 2007; Sokolowski et al., 2011). Furthermore, the early exposure also lead to reduced numbers of mitotic S-phase cells at P21. Is this because acute losses of NSC at P7 remain until P21, or alternatively, do NSC numbers recover but acquire altered proliferation? To determine which cell compartments contribute to reduced total cells and diminished proliferation, P7 pups were injected (sc) with PBS, low MeHg (0.6 μg/g) or high MeHg (5 μg/g) (Figure 1A). Pups were sacrificed at early adolescence (P21) 2 h after BrdU injection and coronal brain sections were immunostained for markers of proliferating cells (Ki67), NSCs (Sox2), intermediate progenitors (Tbr2) and immature neurons (Dcx) (Sokolowski et al., 2013). The Ki67 labeled population (the marker identifies all proliferative cells throughout the cell cycle) was unaffected by this exposure (Figures 1B–E). Further, neither the total Sox2+ NSC population nor the Tbr2+ intermediate precursors were affected by early MeHg exposure (Figures 1F–M). However, rats exposed to low MeHg at P7 had 19% less immature neurons (Dcx) compared to the control group at P21 (Figures 1N–Q), a deficit that likely contributes to the reduction in total cells observed previously. This result supports our recent observation of reduced neurons (~27%) in the GCL 2 weeks after a low MeHg exposure at P7 (Sokolowski et al., 2013), as Dcx precursors ultimately exit the cell cycle to become postmitotic mature neurons.

**Figure 2:**
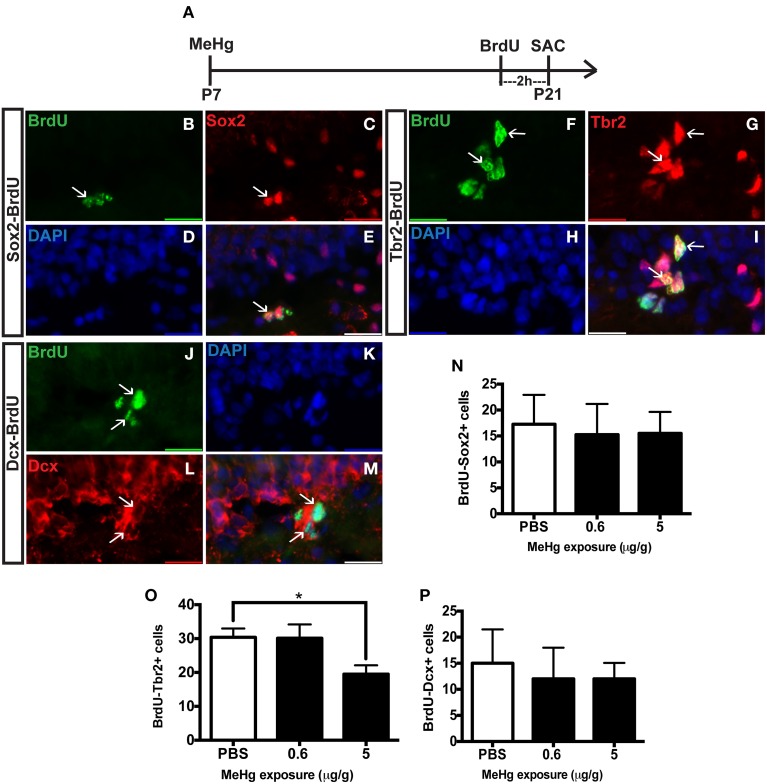
**Early (P7) MeHg exposure leads to a deficit in S-phase of intermediate progenitor cells in the adolescent (P21) hippocampus. (A)** Experimental timeline. **(B–E)** BrdU-Sox2+ double staining in the hilus of the dentate gyrus (DG). Arrows indicate double-positive cells. **(B)** S-phase cells labeled by BrdU (green), **(C)** NSCs labeled with Sox2 (red), **(D)** nuclei labeled with DAPI (blue), **(E)** merged image. **(F–I)** BrdU-Tbr2+ double staining in the SGZ of the DG. **(F)** BrdU (green), **(G)** intermediate progenitor cells labeled by Tbr2 (red), **(H)** DAPI (blue), **(I)** merged image. **(J–M)** BrdU-Dcx+ double staining in the SGZ of the DG. **(J)** BrdU (green), **(K)** DAPI (blue), **(L)** Neuroblasts labeled by Dcx (red), **(M)** merged image. **(N)** Quantification of the average number of BrdU-Sox2+ cells per 5 sections per animal in the hilus. Animals: *n* = 4 per group. Each bar represents mean ± SEM. **(O)** Quantification of the average number of BrdU-Tbr2+ cells per 5 sections per animal in the SGZ. Animals: *n* = 5–8 per group. ^*^p < 0.05 **(P)** Quantification of the average number of BrdU-Dcx+ cells per 5 sections per animal in the SGZ. Animals: *n* = 4 per group.

The observation of an unaffected Ki67 population at P21 upon early MeHg exposure suggests that the acute loss of NSCs at P7 recovers with further development. So then what might account for the reduction of cells in S-phase (BrdU+ cells) observed previously at P21 (Sokolowski et al., 2013)? Potentially, specific stem cell compartments may exhibit reduced progression from G1 into S phase, which we examined using double immunostaining (Figure 2). There was no change in the proportion of early stem cells engaged in S-phase, as reflected by double labeling of BrdU with Sox2 (Figures 2B–E,N) nor of neuroblasts/immature neurons, assessed by BrdU double labeling with Dcx (Figures 2J–M,P). In contrast, high MeHg exposure at P7 elicited a 36% decrease in the intermediate progenitor cells in S-phase at P21 (Figures 2F–I,O). Since there was no change in the number of total Tbr2 cells (Figure 1), early MeHg appears to selectively affect the ability of intermediate progenitors to enter S-phase. This selective change in Tbr2 cell S phase entry may be related to the fact that they are the most rapidly proliferating NSCs in the DG (Hodge et al., 2008). It is notable that the effects of early P7 exposure on P21 cell compartments, Dcx and Tbr2, occur at different exposures.

### Prepubescent rats are vulnerable to MeHg

**Figure 3:**
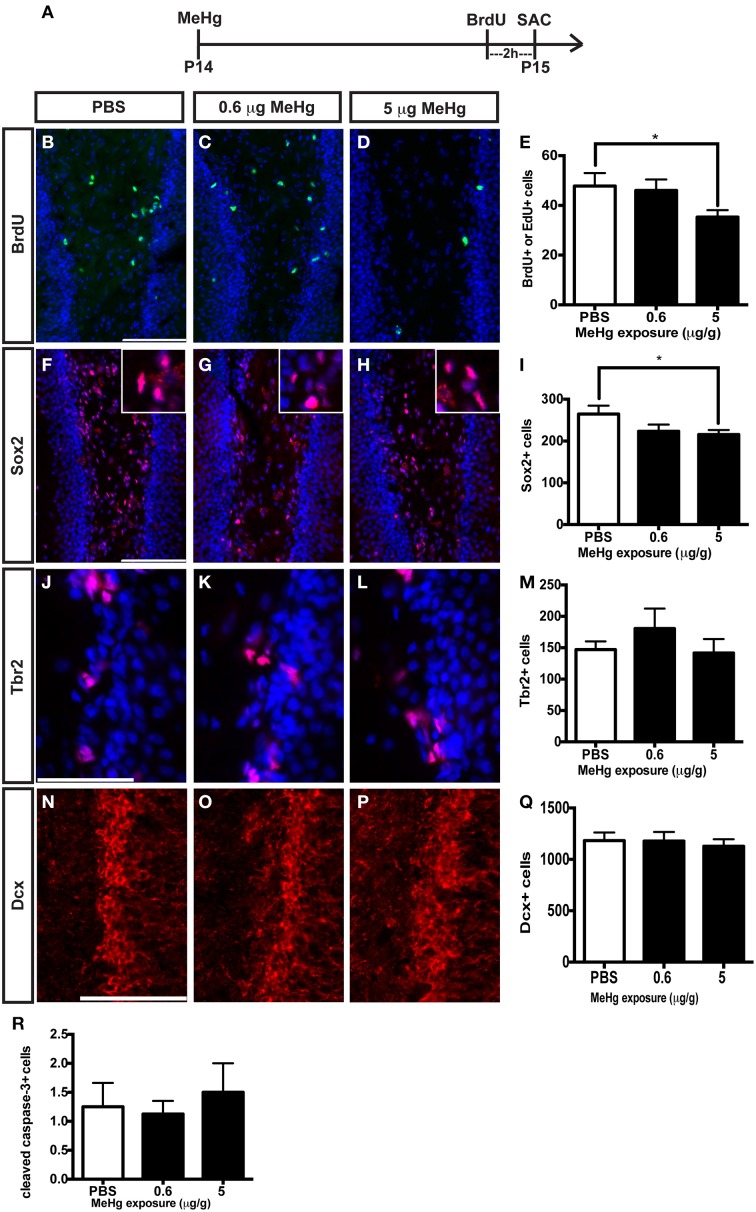
**S-phase cells and NSCs of the prepubescent (P14) hippocampus are vulnerable to acute MeHg exposure. (A)** Experimental timeline. Five sections per brain were counted. **(B–D)** BrdU/EdU staining (green) in the hilus of the DG. Nuclei are labeled with DAPI (blue). **(E)** Quantification of the average number of BrdU+/EdU+ cells per section per animal in the hilus. Animals: *n* = 10 per group. Each bar represents mean ± SEM. Scale bars: 100 μm. **(F–H)** Sox2 staining (red) in the hilus of the DG. **(I)** Quantification of the average number of Sox2+ cells per section per animal in the hilus. Animals: *n* = 4–7 per group. **(J–L)** Tbr2 staining (red) in the hilus of the DG. **(M)** Quantification of the average number of Tbr2+ cells per section per animal in the DG. Animals: *n* = 4–5 per group. **(N–P)** Dcx staining (red) in the GCL of the DG. **(Q)** Quantification of the average number of Dcx+ cells per section per animal in the DG. Animals: *n* = 6 per group. **(R)** Quantification of the average number of cleaved caspase-3+ cells per section per animal in the hilus. Animals: *n* = 8 per group. ^*^p < 0.05.

In our previous studies, we observed that the highly proliferative P7 DG was vulnerable to MeHg, which elicited G1/S cell cycle arrest and acute death of NSCs (Burke et al., 2006; Falluel-Morel et al., 2007; Sokolowski et al., 2011, 2013), indicating that proliferating cells are targets of MeHg. The prepubescent DG has heightened cell proliferation compared to the adult as well, and thus might also be vulnerable. To further explore developmental stage specific vulnerability, we have used the same experimental paradigm as performed at P7, assessing the effects of MeHg on NSC proliferation and apoptosis 24 h after injection, so we can compare outcomes across the ages. P14 pups were injected (sc) with low (0.6 μg/g) or high (5 μg/g) MeHg and sacrificed 24 h later, receiving a BrdU injection 2 h before sacrifice (Figure 3A). High MeHg exposure reduced S-phase cells in the P14 DG hilus by 26% (Figures 3B–E) as well as the number of Sox2+ NSCs by 23% (Figures 3F–I). These similar proportions of reduction may suggest that Sox2+ NSCs are a major cell compartment targeted by MeHg. In contrast, MeHg exposure did not have acute effects on the Tbr2+ intermediate progenitors (Figures 3J–M) even though it is a known target of antimitotic agents (Hodge et al., 2008). To determine if neuroblasts (the last population to incorporate BrdU during neurogenesis, Seri et al., 2004; Encinas et al., 2006) and immature neurons are adversely affected by MeHg, we analyzed Dcx+ cells and did not observe a change in cell number upon either exposure (Figures 3N–Q). This observation suggests that this later stem cell compartment is not sensitive to MeHg at this time. To investigate a potential mechanism producing reductions in BrdU and Sox2 cell populations, we immunostained cells for the apoptotic marker, cleaved caspase-3. However, MeHg did not induce cell death in the hilus at 24 h (Figure 3R). Thus, at this age, cleaved caspase-3 might not be as sensitive a marker for detecting MeHg-induced cell death at 24 h as it was during our P7 exposure studies (Sokolowski et al., 2011), or the time course of apoptosis may differ. In aggregate, these results demonstrate that proliferating hippocampal NSCs at P14 remain vulnerable to MeHg, but primarily at higher levels of exposure.

### MeHg vulnerability is diminished by early adolescence

**Figure 4:**
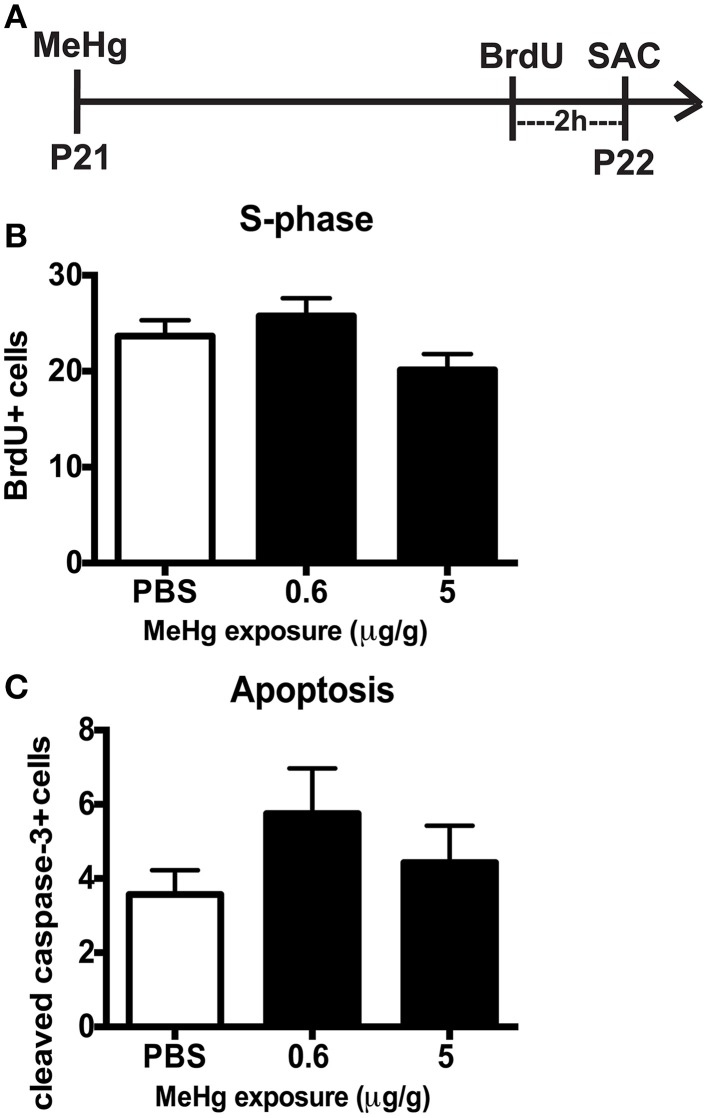
**The adolescent (P21) hippocampus is not vulnerable to 24 h MeHg exposure. (A)** Experimental timeline: P21 rats were given a single sc injection of MeHg (5 μg/g) or vehicle (PBS) at 0 h, and after an ip injection of BrdU at 22 h, were sacrificed at 24 h. Four (4) sections per brain were counted. **(B)** Quantification of the average number of BrdU+ cells per section per animal in the hilus. Animals: *n* = 8–9 per group. Each bar represents mean ± SEM. **(C)** Quantification of the average number of cleaved caspase-3+ cells per section per animal in the hilus. Animals: *n* = 7–8 per group.

By early adolescence (P21), neurogenesis starts to approach the adult basal level (Schlessinger et al., 1975). Using our acute 24 h exposure model, we did not observe a deleterious effect of MeHg at either exposure on the S-phase population nor an increase in apoptosis (Figure 4). There was also no change in the number of Sox2 + cells (PBS = 148 ± 14; MeHg [5 μg/gm] = 116 ± 29; mean Sox2+ cells ± SEM; *p* = 0.3717; *N* = 3/group). We postulated that the exposure time might be too short for the toxicant to have an effect, since expression of neutral amino acid transporters (which are known to import MeHg) in the BBB reduces with age, and the toxicant burden might be reduced or delayed in transit into the hippocampus in older rats (Cornford et al., 1982; Simmons-Willis et al., 2002; Liddelow et al., 2012). Therefore, we assessed an additional exposure time of 48 h and used only high (5 μg/g) MeHg in the single exposure model. Again, there was no effect on S-phase cells (BrdU+ cells: PBS = 22± 1.9; MeHg = 19± 1.2; *p* > 0.1743; *N* = 5−9/group) or apoptosis at either time point (Cleaved caspase-3+ cells: PBS = 6 ± 1.1; MeHg = 5.2 ± 1.1; *p* > 0.6139; *N* = 6−7/group). To determine whether NSCs were affected, we also counted Sox2 labeled cells and observed that they were not negatively impacted by the MeHg insult (Sox2+ cells: PBS = 133 ± 17.5; MeHg = 173 ± 34.1; *p* > 0.3558; *N* = 3/group), suggesting that the adolescent hippocampus is resistant to a developmental MeHg exposure.

### Transfer of MeHg across the BBB does not diminish during the postnatal period

**Figure 5:**
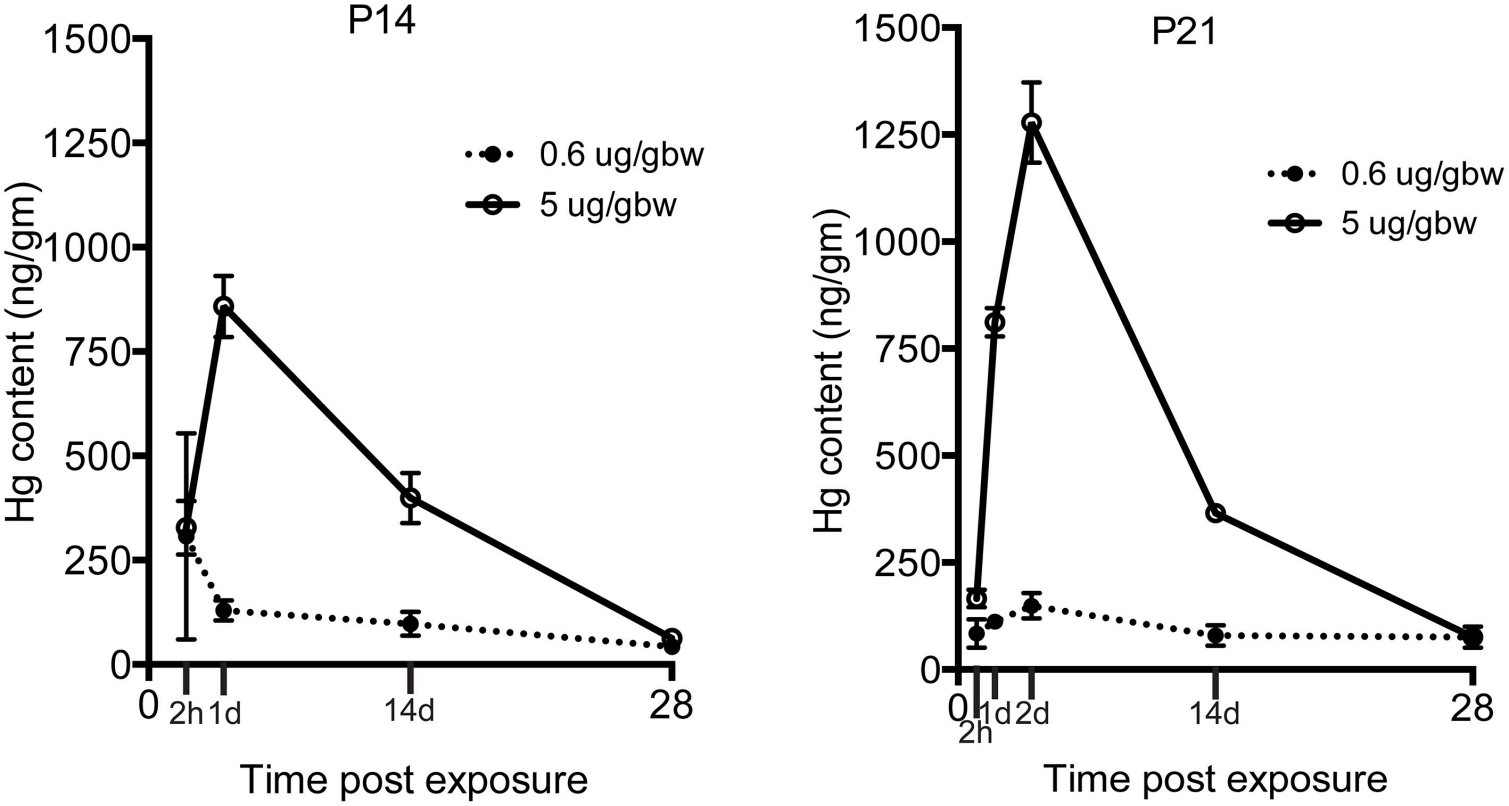
**Transfer of MeHg across the BBB does not diminish during the postnatal period**. Hg concentrations are expressed in nanograms per gram (ng/gm) at time points from 2 h to 4 weeks after two different exposures (0.6 or 5 μg/MeHg) in two age groups (P14 and P21). Control samples were obtained at all-time points and pooled for each age cohort for analysis (P14 control = <28 ng/gm, P21 control = <26 ng/gm). After exposure to 0.6 and 5 μg/MeHg at both ages, significant levels of Hg were observed at all-time points.

Because the same MeHg exposures had diminishing effects on hippocampal neurogenesis at older ages, we wondered whether transfer might be restricted with development. To examine this issue we injected both low and high dose MeHg sc as before and then dissected the hippocampus at 2 and 24 h, as well as at 2 and 4 weeks, the same intervals assessed previously for P7 rats (Figure 5) (Sokolowski et al., 2013). At both ages (and exposures), there was rapid accumulation of Hg in the hippocampus already at 2 h (327 ng/gm at P14; 166 ng/gm at P21; 5 μg/gm MeHg). By 24 h, there was >800 ng/gm Hg for the high exposure at both ages, a level that was 6–9 times greater than the lower exposure. By 2 weeks, the levels were ~400 ng/gm for the high exposure for both ages, a level that was about 4 times greater than the low exposure. By 4 weeks, the majority of Hg, >90% of the early level, had dissipated, though there were minor residual levels, which contrasts with P7 injections that exhibited no detectable Hg at 4 weeks after injection (Sokolowski et al., 2013).

## Discussion

### Effects of perinatal MeHg on adolescent neurogenesis

We found that early exposure at P7 has lasting effects on adolescent (P21) neurogenesis since fewer intermediate progenitor cells (Tbr2) were undergoing S-phase (BrdU), and there were deficits in the total number of immature neurons (Dcx), though these different effects reflected distinct exposures. In contrast, there was no long-term effect of acute P7 exposure on the total proliferating cell fraction (Ki67) or NSCs (Sox2) at P21. Thus, the Sox2 NSC population, which is the majority of proliferating cells in the DG (Encinas and Sierra, 2012), is only affected by MeHg acutely, but at longer terms it apparently recovers. When we used this same P7 exposure model in previous studies, we observed a reduction in the S-phase cell fraction at P21. This apparent discrepancy between the adolescent Ki67 and BrdU labeling suggests that early MeHg exposure prevents or delays a subset of P21 precursor cells from entering S-phase, a change in long-term precursor proliferative activity. Previously, we demonstrated that acute P7 MeHg exposure arrests proliferating cells at the G1/S checkpoint, leading to cell death within 24 h (Burke et al., 2006; Falluel-Morel et al., 2007). In the current study, we find that 2 weeks after high MeHg exposure the proportion of intermediate progenitor cells engaged in S-phase (BrdU-Tbr2+) is reduced, suggesting that they are the main population displaying deficits in S-phase (Table 1). We speculate that the cells that survive the acute initial insult either fail to enter S-phase, or continue to proliferate, albeit more slowly because of the continued presence of MeHg. In future studies we may test for a slower cell cycle at P21 by injecting multiple thymidine analogs such as CldU and IdU at different intervals after MeHg exposure, determining cell cycle lengths of different cell populations using triple immunofluorescence (Vega and Peterson, 2005; Tanaka et al., 2011; Brandt et al., 2012; Farioli-Vecchioli et al., 2014).

**Table 1:** **Summary of previous and current results**.

**Acute MeHg at P7**			**Long-term MeHg effects at P21**	
**EFFECTS OF EARLY MeHg EXPOSURE ON HIPPOCAMPUS**				
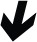 Cell cycle progression^a,b^			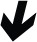 DG volume and cell and neuron numbers ^b,c^	
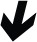 S-phase cells^a,b,c^			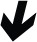 S-phase cells ^c^	
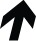 Apoptosis of neural stem cells^c^ (caspase-3-Sox2/nestin)			**— Proliferating cells (Ki67)** 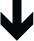 **Intermediate progenitor S-phase cells (BrdU-Tbr2)** 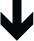 **Immature Dcx neurons** 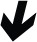 Spatial learning and memory^b,c^	
**Age**	**S-phase cells (BrdU)**	**Early precursors (Sox2)**	**Intermediate progenitors (Tbr2)**	**Immature neurons (Dcx)**
**ACUTE MeHg EFFECTS BY AGE**				
P7	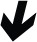 ^a,b,c^	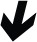 ^c^		
P14	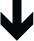	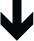	No effect	No effect
P21	No effect	No effect	No effect	No effect

*These data demonstrate the acute and long-term effects of MeHg exposure on specific hippocampal cell compartments, which are implicated in the presentation of behavioral deficits in early adulthood. Previous data is italicized; current data is bolded*.

a *Data from Burke et al. (2006)*.

b *Data from Falluel-Morel et al. (2007)*.

c *Data from Sokolowski et al. (2013)*.

While P7 MeHg exposure had long-term effects on the Tbr2 and Dcx subpopulations at P21, the former cells were vulnerable to only high exposure whereas the latter were decreased with low exposure only. It has long been known that MeHg exposure has complex effects on cell biological processes, which may exhibit multiphasic responses (Hare and Atchison, 1995). This is likely due to MeHg affecting many different molecular targets as concentrations are varied. As a divalent cation, MeHg can (1) compete with Ca^2+^ in intracellular signaling, (2) lead to reactive oxygen species (ROS) generation that affects mitochondrial function, (3) alter gene expression, and (4) bind to unbiquitous sulfhydryl groups on cysteines and methionines in diverse proteins, like tubulin (Limke et al., 2004; Falluel-Morel et al., 2007; Ceccatelli et al., 2010). Thus, it may be expected that uniform changes in biological outcomes will not be seen with increasing exposures, but rather qualitative differences may occur. While not yet defined, the effects of P7 low MeHg exposure on later P21 Dcx cell number may fall into this category, whereas higher early exposure may no longer change the Dcx population due to actions at other sites, but may now affect the most rapidly proliferating Tbr2 population. To examine this issue we might assess a range of acute molecular responses after low and high MeHg exposure at P7, such as Ca^2+^ levels, mitochondrial membrane potential, Ca^2+^ dependent signals [calpain, PKC], cell cycle regulators and developmental regulatory genes (Sox2, Tbr2, Lhx2, NeuroD, Prox1). For example, low exposure might affect Prox1, which regulates the transition from proliferative intermediate Tbr2+ precursor to postmitotic Dcx-expressing immature neurons (Stergiopoulos et al., 2014), whereas high exposures may directly affect cell cycle regulators such as cyclins and/or CDK inhibitors (Tury et al., 2012). Furthermore, some of these changes may only occur in specific cell compartments, such as those expressing distinct stage specific transcription factors, a direction for future study.

### MeHg vulnerability diminishes by early adolescence

Our second major finding is that the developmental window of vulnerability to MeHg in the hippocampus ends between prepubescence and adolescence. Until recently, our studies on acute MeHg exposure were performed in P7 rats, which is a highly sensitive developmental period in rodents that corresponds to the third trimester of human gestation. This current study demonstrates that the prepubescent rat hippocampus is vulnerable to MeHg (Table 1). Similar to the P7 rat, acute exposure at P14 decreased the number of proliferating (BrdU+) cells and NSCs (Sox2) after 24 h, though the magnitude of change was smaller. This suggests that Sox2+ precursors are consistent targets of acute MeHg exposure during early postnatal life. However, there was no sign of vulnerability in the P21 hippocampus at 24 or 48 h postexposure. Furthermore, the proportion of cleaved caspase-3 labeled cells, which we used previously as a sensitive marker of acute MeHg toxicity at P7 (Sokolowski et al., 2013), was not increased upon exposure at these older ages. One possibility is that the kinetics of cell death might be different at these ages, with the peak of apoptosis occurring earlier or later than 24 h.

Given that the overall magnitude of the neurotoxic effect of MeHg at P14 was smaller than at P7, and entirely absent at P21, we wondered whether this may be due to changes in the BBB. During fetal development, tight junctions, which provide a structural barrier against xenobiotics, are formed and many transporters involved in influx and efflux mechanisms are expressed (Saunders and Mollgard, 1984). Further, there is evidence that the expression levels of the neutral amino acid transporters, which are a known conduit for MeHg (Simmons-Willis et al., 2002), are higher in the developing BBB than in the adult (Cornford et al., 1982; Liddelow et al., 2012). Thus, it was perhaps surprising that the transfer of Hg into the hippocampus at both P14 and P21 was robust, accumulating already at 2 h, and increasing for 24 h (and in the case of P21, even further at 48 h). These kinetics are very similar to our previous results performed at P7 (Sokolowski et al., 2013). Two weeks after the injections, levels in the hippocampus were approximately half that at 24 h, and by 4 weeks, >90% of the Hg was gone, suggesting effective mechanisms for clearance at both ages. Thus, what explains the diminished effects at older ages? We suspect both the NSCs themselves and neighboring cells play major roles in neuroprotection. Regional expression of antioxidants such as glutathione might confer additional protection in the brain. For example, astrocytes (which are a significant source of glutathione precursors, Allen et al., 2002) isolated from the cerebellum have lower glutathione capacity and are therefore more sensitive to MeHg exposure than astrocytes from the cortex (Kaur et al., 2007). The increasing presence of glia that are generated during the postnatal period in rat (Bignami and Dahl, 1973) might also affect the MeHg burden to NSCs. Microglia and astrocytes secrete neurotrophic factors in the NSC niche, which might provide protection against a MeHg insult (Sierra et al., 2014). When cultured with neurons, astroctyes increase their uptake of MeHg, leading to reduced neuronal MeHg uptake and mitochondrial dysfunction (Morken et al., 2005). In summary, these results provide new knowledge of an expanded period of neurodevelopmental vulnerability (Table 1) that diminishes secondary to changes in NSCs and surrounding tissues.

MeHg is one of the numerous environmental agents that cause developmental neurotoxicity by altering NSC proliferation. Other agents include ionizing radiation and methylazoxymethanol (MAM), which are antimitotic, and ethanol, chlorpyrifos (organophosphorus pesticide), and lead (Rice and Barone, 2000; Fox et al., 2012). Antimitotic agents are most detrimental during periods (and in areas) of high cell proliferation in the brain. For example, the ionizing radiation used for brain cancer therapy has been shown to reduce neurogenesis and impair hippocampal-dependent behaviors in adult rats (Nokia et al., 2012). Excessive environmental exposure to manganese (an essential trace element which is neurotoxic at higher concentrations) pre- and postnatally kills immature granule neurons in mice (Wang et al., 2012), and is associated with impaired cognitive performance in children (Roels et al., 2012). Recent studies have linked the development of child and adult-onset metabolic, neurological, and neurodegenerative diseases to toxicant exposures during gestation and the perinatal period (Fox et al., 2012). Our current observation of decreased adolescent neurogenesis and learning and memory deficits (Falluel-Morel et al., 2007; Sokolowski et al., 2013) after perinatal MeHg exposure in rats is not surprising, considering that various epidemiological studies show an association between prenatal exposure and cognitive deficits (Grandjean et al., 1997; Clarkson and Magos, 2006). Pre- and postnatal lead exposure is linked to childhood obesity (Fox et al., 2012). The antimitotic agent MAM is found in the cycad seed, which is widely consumed in Guam, Kii Peninsula (Japan), and West Papua. Gestational MAM exposure is implicated in western Pacific amyotrophic lateral sclerosis and Parkinson-dementia complex (ALS–PDC) in these populations (Kisby and Spencer, 2011). Prenatal MAM exposure has also been used to induce positive and negative symptoms of schizophrenia in rats (Gourevitch et al., 2004; Flagstad et al., 2005; Hazane et al., 2009). Our findings in this study corroborate that neurogenesis is a relevant process to study when identifying developmental neurotoxicants, and that vulnerability will likely be stage-specific.

Toxicants that contribute to neurological disorders and disease pose not only a public health risk, but also an economic risk because possessing a reduced IQ leads to lower productivity and the associated mental health treatment costs are increasingly expensive (Trasande et al., 2005). Knowing the extent of developmental vulnerability to common neurotoxicants will help the scientific community and regulatory agencies to properly assess safe environmental levels of substances which have unknown toxicity.

### Conflict of interest statement

The authors declare that the research was conducted in the absence of any commercial or financial relationships that could be construed as a potential conflict of interest.

## References

[B1] Allen J. W. Shanker G. Tan K. H. Aschner M. (2002). The consequences of methylmercury exposure on interactive functions between astrocytes and neurons. Neurotoxicology 23, 755–759. 10.1016/S0161-813X(01)00076-6 12520765

[B2] Altman J. Bayer S. A. (1990). Migration and distribution of two populations of hippocampal granule cell precursors during the perinatal and postnatal periods. J. Comp. Neurol. 301, 365–381. 2262596 10.1002/cne.903010304

[B3] Amin-Zaki L. Elhassani S. Majeed M. A. Clarkson T. W. Doherty R. A. Greenwood M. R.. (1976). Perinatal methylmercury poisoning in Iraq. Am. J. Dis. Child. 130, 1070–1076. 973609 10.1001/archpedi.1976.02120110032004

[B4] Bignami A. Dahl D. (1973). Differentiation of astrocytes in the cerebellar cortex and the pyramidal tracts of the newborn rat. An immunofluorescence study with antibodies to a protein specific to astrocytes. Brain Res. 49, 393–402. 4578563 10.1016/0006-8993(73)90430-7

[B5] Brandt M. D. Hubner M. Storch A. (2012). Brief report: adult hippocampal precursor cells shorten S-phase and total cell cycle length during neuronal differentiation. Stem Cells 30, 2843–2847. 10.1002/stem.1244 22987479

[B6] Burke K. Cheng Y. Li B. Petrov A. Joshi P. Berman R.. (2006). Methylmercury elicits rapid inhibition of cell proliferation in the developing brain and decreases cell cycle regulator, cyclin E. Neurotoxicology 27, 970–981. 10.1016/j.neuro.2006.09.001 17056119 PMC2013736

[B7] Ceccatelli S. Dare E. Moors M. (2010). Methylmercury-induced neurotoxicity and apoptosis. Chem. Biol. Interact. 188, 301–308. 10.1016/j.cbi.2010.04.007 20399200

[B8] Cheng Y. Black I. B. Dicicco-Bloom E. (2002). Hippocampal granule neuron production and population size are regulated by levels of bFGF. Eur. J. Neurosci. 15, 3–12. 10.1046/j.0953-816x.2001.01832.x 11860501

[B9] Choi B. H. Lapham L. W. Amin-Zaki L. Saleem T. (1978). Abnormal neuronal migration, deranged cerebral cortical organization, and diffuse white matter astrocytosis of human fetal brain: a major effect of methylmercury poisoning *in utero*. J. Neuropathol. Exp. Neurol. 37, 719–733. 739273 10.1097/00005072-197811000-00001

[B10] Clarkson T. Magos L. (2006). The toxicology of mercury and its chemical compounds. Crit. Rev. Toxicol. 36, 609–662. 10.1080/10408440600845619 16973445

[B11] Cornford E. M. Braun L. D. Oldendorf W. H. (1982). Developmental modulations of blood-brain barrier permeability as an indicator of changing nutritional requirements in the brain. Pediatr. Res. 16, 324–328. 7079003 10.1203/00006450-198204000-00017

[B12] Crump K. S. Kjellstrom T. Shipp A. M. Silvers A. Stewart A. (1998). Influence of prenatal mercury exposure upon scholastic and psychological test performance: benchmark analysis of a New Zealand cohort. Risk Anal. 18, 701–713. 9972579 10.1023/b:rian.0000005917.52151.e6

[B13] Davidson P. Cory-Slechta D. Thurston S. Huang L.-S. Shamlaye C. Gunzler D.. (2011). Fish consumption and prenatal methylmercury exposure: cognitive and behavioral outcomes in the main cohort at 17 years from the Seychelles child development study. Neurotoxicology 32, 711–717. 10.1016/j.neuro.2011.08.003 21889535 PMC3208775

[B14] Debes F. Budtz-Jorgensen E. Weihe P. White R. F. Grandjean P. (2006). Impact of prenatal methylmercury exposure on neurobehavioral function at age 14 years. Neurotoxicol. Teratol. 28, 536–547. 10.1016/j.ntt.2006.02.005 17067778

[B15] Deng W. Aimone J. Gage F. (2010). New neurons and new memories: how does adult hippocampal neurogenesis affect learning and memory? Nature reviews. Neuroscience 11, 339–350. 10.1038/nrn2822 20354534 PMC2886712

[B16] Eisch A. J. Cameron H. A. Encinas J. M. Meltzer L. A. Ming G. L. Overstreet-Wadiche L. S. (2008). Adult neurogenesis, mental health, and mental illness: hope or hype? J. Neurosci. 28, 11785–11791. 10.1523/JNEUROSCI.3798-08.2008 19005040 PMC2793333

[B17] Encinas J. Vaahtokari A. Enikolopov G. (2006). Fluoxetine targets early progenitor cells in the adult brain. Proc. Natl. Acad. Sci. U.S.A. 103, 8233–8238. 10.1073/pnas.0601992103 16702546 PMC1461404

[B18] Encinas J. M. Sierra A. (2012). Neural stem cell deforestation as the main force driving the age-related decline in adult hippocampal neurogenesis. Behav. Brain Res. 227, 433–439. 10.1016/j.bbr.2011.10.010 22019362

[B19] Falluel-Morel A. Lin L. Sokolowski K. McCandlish E. Buckley B. Dicicco-Bloom E. (2012). N-acetyl cysteine treatment reduces mercury-induced neurotoxicity in the developing rat hippocampus. J. Neurosci. Res. 90, 743–750. 10.1002/jnr.22819 22420031 PMC3306130

[B20] Falluel-Morel A. Sokolowski K. Sisti H. M. Zhou X. Shors T. J. Dicicco-Bloom E. (2007). Developmental mercury exposure elicits acute hippocampal cell death, reductions in neurogenesis, and severe learning deficits during puberty. J. Neurochem. 103, 1968–1981. 10.1111/j.1471-4159.2007.04882.x 17760861 PMC3363963

[B21] Farioli-Vecchioli S. Mattera A. Micheli L. Ceccarelli M. Leonardi L. Saraulli D.. (2014). Running rescues defective adult neurogenesis by shortening the length of the cell cycle of neural stem and progenitor cells. Stem Cells 32, 1968–1982. 10.1002/stem.1679 24604711

[B22] Flagstad P. Glenthoj B. Y. Didriksen M. (2005). Cognitive deficits caused by late gestational disruption of neurogenesis in rats: a preclinical model of schizophrenia. Neuropsychopharmacology 30, 250–260. 10.1038/sj.npp.1300625 15578007

[B23] Fox D. A. Grandjean P. De Groot D. Paule M. G. (2012). Developmental origins of adult diseases and neurotoxicity: epidemiological and experimental studies. Neurotoxicology 33, 810–816. 10.1016/j.neuro.2011.12.016 22245043 PMC3657611

[B24] Gourevitch R. Rocher C. Le Pen G. Krebs M. O. Jay T. M. (2004). Working memory deficits in adult rats after prenatal disruption of neurogenesis. Behav. Pharmacol. 15, 287–292. 10.1097/01.fbp.0000135703.48799.71 15252279

[B25] Grandjean P. Weihe P. White R. Debes F. Araki S. Yokoyama K.. (1997). Cognitive deficit in 7-year-old children with prenatal exposure to methylmercury. Neurotoxicol. Teratol. 19, 417–428. 9392777 10.1016/s0892-0362(97)00097-4

[B26] Harada M. Akagi H. Tsuda T. Kizaki T. Ohno H. (1999). Methylmercury level in umbilical cords from patients with congenital Minamata disease. Sci. Total Environ. 234, 59–62. 10507148 10.1016/s0048-9697(99)00255-7

[B27] Hare M. F. Atchison W. D. (1995). Nifedipine and tetrodotoxin delay the onset of methylmercury-induced increase in [Ca^2+^]i in NG108-15 cells. Toxicol. Appl. Pharmacol. 135, 299–307. 8545840 10.1006/taap.1995.1236

[B28] Hazane F. Krebs M. O. Jay T. M. Le Pen G. (2009). Behavioral perturbations after prenatal neurogenesis disturbance in female rat. Neurotox. Res. 15, 311–320. 10.1007/s12640-009-9035-z 19384565

[B29] Hodge R. Kowalczyk T. Wolf S. Encinas J. Rippey C. Enikolopov G.. (2008). Intermediate progenitors in adult hippocampal neurogenesis: Tbr2 expression and coordinate regulation of neuronal output. J. Neurosci. 28, 3707–3717. 10.1523/JNEUROSCI.4280-07.2008 18385329 PMC6671086

[B30] Kaur P. Aschner M. Syversen T. (2007). Role of glutathione in determining the differential sensitivity between the cortical and cerebellar regions towards mercury-induced oxidative stress. Toxicology 230, 164–177. 10.1016/j.tox.2006.11.058 17169475

[B31] Kempermann G. (2011). Adult Neurogenesis 2: Stem Cells and Neuronal Development in the Adult Brain. New York, NY: Oxford University Press.

[B32] Kempermann G. Jessberger S. Steiner B. Kronenberg G. (2004). Milestones of neuronal development in the adult hippocampus. Trends Neurosci. 27, 447–452. 10.1016/j.tins.2004.05.013 15271491

[B33] Kisby G. Spencer P. (2011). Is neurodegenerative disease a long-latency response to early-life genotoxin exposure? Int. J. Environ. Res. Public Health 8, 3889–3921. 10.3390/ijerph8103889 22073019 PMC3210588

[B34] Kjellstrom T. Kennedy P. Wallis S. Mantell C. (1986). Physical and Mental Development of Children with Prenatal Exposure to Mercury from Fish. Stage 1: Preliminary Tests at Age 4, Report 3080. Solna: National Swedish Environmental Protection Board.

[B35] Kjellstrom T. Kennedy P. Wallis S. Stewart A. Friberg L. Lind B. (1989). Physical and Mental Development of Children with Prenatal Exposure to Mercury from Fish. Stage II: Interviews and Psychological Tests at Age 6, Report 3642. Solna: National Swedish Environmental Protection Board.

[B36] Liddelow S. A. Temple S. Mollgard K. Gehwolf R. Wagner A. Bauer H.. (2012). Molecular characterisation of transport mechanisms at the developing mouse blood-CSF interface: a transcriptome approach. PLoS ONE 7:e33554. 10.1371/journal.pone.0033554 22457777 PMC3310074

[B37] Limke T. L. Bearss J. J. Atchison W. D. (2004). Acute exposure to methylmercury causes Ca^2+^ dysregulation and neuronal death in rat cerebellar granule cells through an M3 muscarinic receptor-linked pathway. Toxicol. Sci. 80, 60–68. 10.1093/toxsci/kfh131 15141107

[B38] Matsumoto H. Koya G. Takeuchi T. (1965). Fetal Minamata disease. A neuropathological study of two cases of intrauterine intoxication by a methyl mercury compound. J. Neuropathol. Exp. Neurol. 24, 563–574. 5890913

[B39] Morken T. S. Sonnewald U. Aschner M. Syversen T. (2005). Effects of methylmercury on primary brain cells in mono- and co-culture. Toxicol. Sci. 87, 169–175. 10.1093/toxsci/kfi227 15958655

[B40] Myers G. J. Davidson P. W. Cox C. Shamlaye C. F. Tanner M. A. Choisy O.. (1995a). Neurodevelopmental outcomes of Seychellois children sixty-six months after *in utero* exposure to methylmercury from a maternal fish diet: pilot study. Neurotoxicology 16, 639–652. 8714869

[B41] Myers G. J. Davidson P. W. Cox C. Shamlaye C. F. Tanner M. A. Marsh D. O.. (1995b). Summary of the Seychelles child development study on the relationship of fetal methylmercury exposure to neurodevelopment. Neurotoxicology 16, 711–716. 8714875

[B42] Myers G. J. Marsh D. O. Davidson P. W. Cox C. Shamlaye C. F. Tanner M.. (1995c). Main neurodevelopmental study of Seychellois children following *in utero* exposure to methylmercury from a maternal fish diet: outcome at six months. Neurotoxicology 16, 653–664. 8714870

[B43] NAS. (2000). National Research Council (US) Committee on the Toxicological Effects of Methylmercury. Washington, DC: The National Academies Press. 25077280

[B44] Nokia M. S. Anderson M. L. Shors T. J. (2012). Chemotherapy disrupts learning, neurogenesis and theta activity in the adult brain. Eur. J. Neurosci. 36, 3521–3530. 10.1111/ejn.12007 23039863 PMC3523213

[B45] Philbert M. A. Billingsley M. L. Reuhl K. R. (2000). Mechanisms of injury in the central nervous system. Toxicol. Pathol. 28, 43–53. 10.1177/019262330002800107 10668990

[B46] Rasband W. (1997–2012). ImageJ. Bethesda, MD: U.S. National Institutes of Health.

[B47] Rice D. Barone S. (2000). Critical periods of vulnerability for the developing nervous system: evidence from humans and animal models. Environ. Health Perspect. 108(Suppl. 3), 511–533. 10.1289/ehp.00108s3511 10852851 PMC1637807

[B48] Rodier P. M. Aschner M. Sager P. R. (1984). Mitotic arrest in the developing CNS after prenatal exposure to methylmercury. Neurobehav. Toxicol. Teratol. 6, 379–385. 6514102

[B49] Roels H. A. Bowler R. M. Kim Y. Claus Henn B. Mergler D. Hoet P.. (2012). Manganese exposure and cognitive deficits: a growing concern for manganese neurotoxicity. Neurotoxicology 33, 872–880. 10.1016/j.neuro.2012.03.009 22498092 PMC3839941

[B50] Saunders N. R. Mollgard K. (1984). Development of the blood-brain barrier. J. Dev. Physiol. 6, 45–57. 6368666

[B51] Schlessinger A. R. Cowan W. M. Gottlieb D. I. (1975). An autoradiographic study of the time of origin and the pattern of granule cell migration in the dentate gyrus of the rat. J. Comp. Neurol. 159, 149–175. 10.1002/cne.901590202 1112911

[B52] Seri B. García-Verdugo J. Collado-Morente L. McEwen B. Alvarez-Buylla A. (2004). Cell types, lineage, and architecture of the germinal zone in the adult dentate gyrus. J. Comp. Neurol. 478, 359–378. 10.1002/cne.20288 15384070

[B53] Sierra A. Beccari S. Diaz-Aparicio I. Encinas J. Comeau S. Tremblay M.-È. (2014). Surveillance, phagocytosis, and inflammation: how never-resting microglia influence adult hippocampal neurogenesis. Neural Plast. 2014:610343. 10.1155/2014/610343 24772353 PMC3977558

[B54] Sierra A. Encinas J. Deudero J. Chancey J. Enikolopov G. Overstreet-Wadiche L.. (2010). Microglia shape adult hippocampal neurogenesis through apoptosis-coupled phagocytosis. Cell Stem Cell 7, 483–495. 10.1016/j.stem.2010.08.014 20887954 PMC4008496

[B55] Simmons-Willis T. A. Koh A. S. Clarkson T. W. Ballatori N. (2002). Transport of a neurotoxicant by molecular mimicry: the methylmercury-L-cysteine complex is a substrate for human L-type large neutral amino acid transporter (LAT) 1 and LAT2. Biochem. J. 367, 239–246. 10.1042/BJ20020841 12117417 PMC1222880

[B56] Sokolowski K. Falluel-Morel A. Zhou X. Dicicco-Bloom E. (2011). Methylmercury (MeHg) elicits mitochondrial-dependent apoptosis in developing hippocampus and acts at low exposures. Neurotoxicology 32, 535–544. 10.1016/j.neuro.2011.06.003 21741406 PMC3256128

[B57] Sokolowski K. Obiorah M. Robinson K. McCandlish E. Buckley B. Dicicco-Bloom E. (2013). Neural stem cell apoptosis after low-methylmercury exposures in postnatal hippocampus produce persistent cell loss and adolescent memory deficits. Dev. Neurobiol. 73, 936–949. 10.1002/dneu.22119 23959606 PMC3874131

[B58] Stergiopoulos A. Elkouris M. Politis P. K. (2014). Prospero-related homeobox 1 (Prox1) at the crossroads of diverse pathways during adult neural fate specification. Front. Cell. Neurosci. 8:454. 10.3389/fncel.2014.00454 25674048 PMC4306308

[B59] Tanaka R. Tainaka M. Ota T. Mizuguchi N. Kato H. Urabe S.. (2011). Accurate determination of S-phase fraction in proliferative cells by dual fluorescence and peroxidase immunohistochemistry with 5-bromo-2'-deoxyuridine (BrdU) and Ki67 antibodies. J. Histochem. Cytochem. 59, 791–798. 10.1369/0022155411411090 21551319 PMC3261604

[B60] Trasande L. Landrigan P. J. Schechter C. (2005). Public health and economic consequences of methyl mercury toxicity to the developing brain. Environ. Health Perspect. 113, 590–596. 10.1289/ehp.7743 15866768 PMC1257552

[B61] Tury A. Mairet-Coello G. Dicicco-Bloom E. (2012). The multiple roles of the cyclin-dependent kinase inhibitory protein p57(KIP2) in cerebral cortical neurogenesis. Dev. Neurobiol. 72, 821–842. 10.1002/dneu.20999 22076965

[B62] Vega C. J. Peterson D. A. (2005). Stem cell proliferative history in tissue revealed by temporal halogenated thymidine analog discrimination. Nat. Methods 2, 167–169. 10.1038/nmeth741 15782184

[B63] Wagner J. P. Black I. B. Dicicco-Bloom E. (1999). Stimulation of neonatal and adult brain neurogenesis by subcutaneous injection of basic fibroblast growth factor. J. Neurosci. 19, 6006–6016. 10407038 10.1523/JNEUROSCI.19-14-06006.1999 PMC6783097

[B64] Wang L. Ohishi T. Shiraki A. Morita R. Akane H. Ikarashi Y.. (2012). Developmental exposure to manganese chloride induces sustained aberration of neurogenesis in the hippocampal dentate gyrus of mice. Toxicol. Sci. 127, 508–521. 10.1093/toxsci/kfs110 22407947

[B65] Weiss B. Clarkson T. W. Simon W. (2002). Silent latency periods in methylmercury poisoning and in neurodegenerative disease. Environ. Health Perspect. 110(Suppl. 5), 851–854. 10.1289/ehp.02110s5851 12426145 PMC1241259

